# Supplementary material for: The computational relationship between reinforcement learning, social inference, and paranoia
Source: PLoS Comput Biol. 2022 Jul 25;18(7):e1010326. doi: 10.1371/journal.pcbi.1010326 (PMC9352206; doi:10.1371/journal.pcbi.1010326)
Supplement: S1 Fig — Top panel: relationship of paranoia and ICAR total score with the proportion of correct cards chosen in each block. Bottom panel: Sum of each chosen card by paranoia and ICAR total score for each block. In Block 1, Card 1 was the optimal card to choose with an 80/20 probability of reward. In Block 2, Card 3 was the optimal card to choose, with 80/20 probability of reward. (DOCX) [file pcbi.1010326.s001.docx]

**
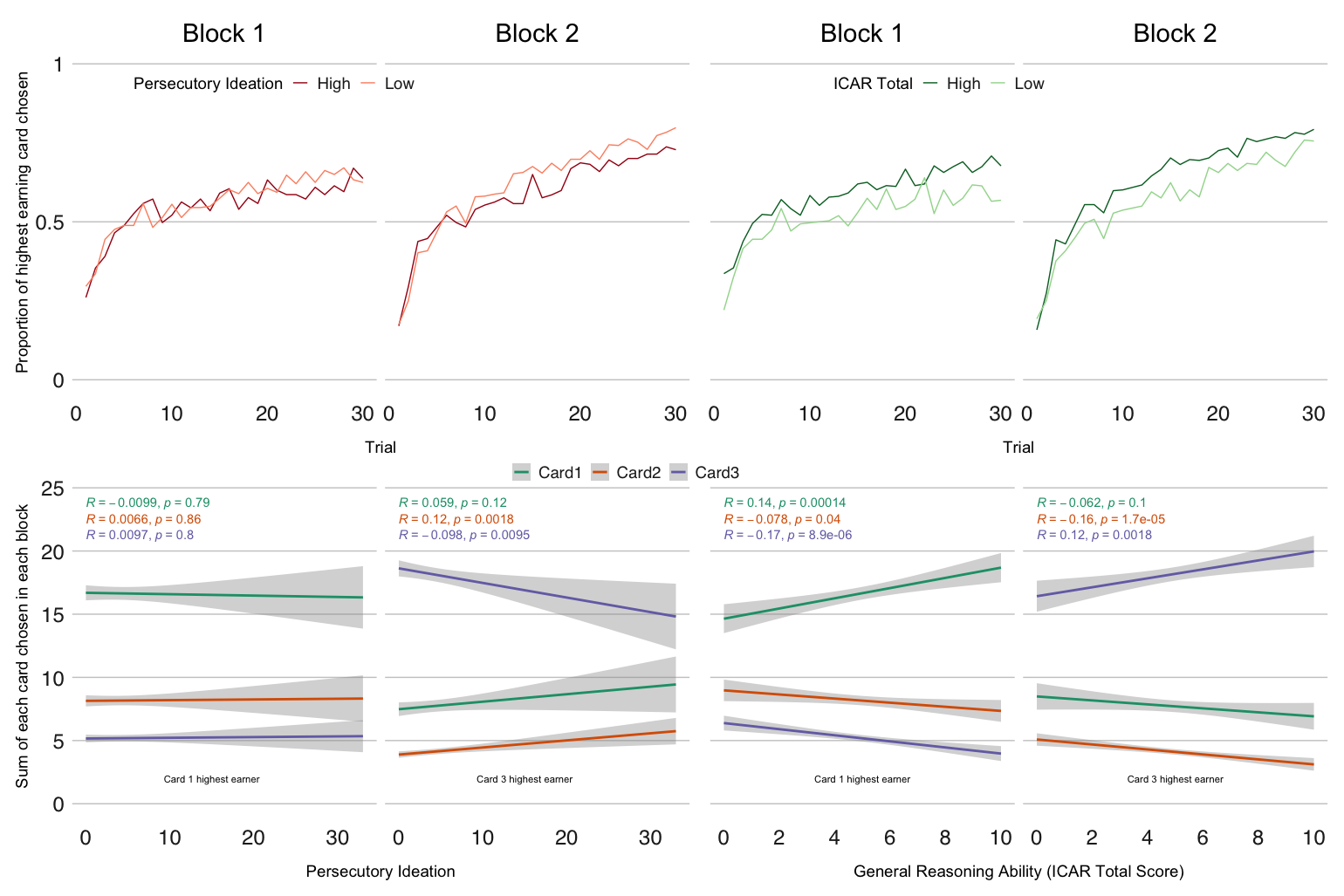
**

**Figure S1: Behaviour of the participants in the probabilistic reasoning task.**

Top panel: relationship of paranoia and ICAR total score with the proportion of correct cards chosen in each block. Bottom panel: Sum of each chosen card by paranoia and ICAR total score for each block. In Block 1, Card 1 was the optimal card to choose with an 80/20 probability of reward. In Block 2, Card 3 was the optimal card to choose, with 80/20 probability of reward.
