# Supplementary material for: The computational relationship between reinforcement learning, social inference, and paranoia
Source: PLoS Comput Biol. 2022 Jul 25;18(7):e1010326. doi: 10.1371/journal.pcbi.1010326 (PMC9352206; doi:10.1371/journal.pcbi.1010326)
Supplement: S2 Fig — In Block 1, Card 1 was the optimal choice with an 80/20 probability of reward. In Block 2, Card 3 was the optimal choice, with 80/20 probability of reward. This graph demonstrates that those with higher paranoia were significantly and more consistently likely to choose the suboptimal 20/80 card (Card 2) in block two, and significantly less and more consistently likely to ignore the optimal card (Card 3) in block 2. However, those with higher paranoia were still able to learn which was the more optimal card by the end of block 2. * = p<0.05, ** = p<0.01 *** = p<0.001. (DOCX) [file pcbi.1010326.s002.docx]

**
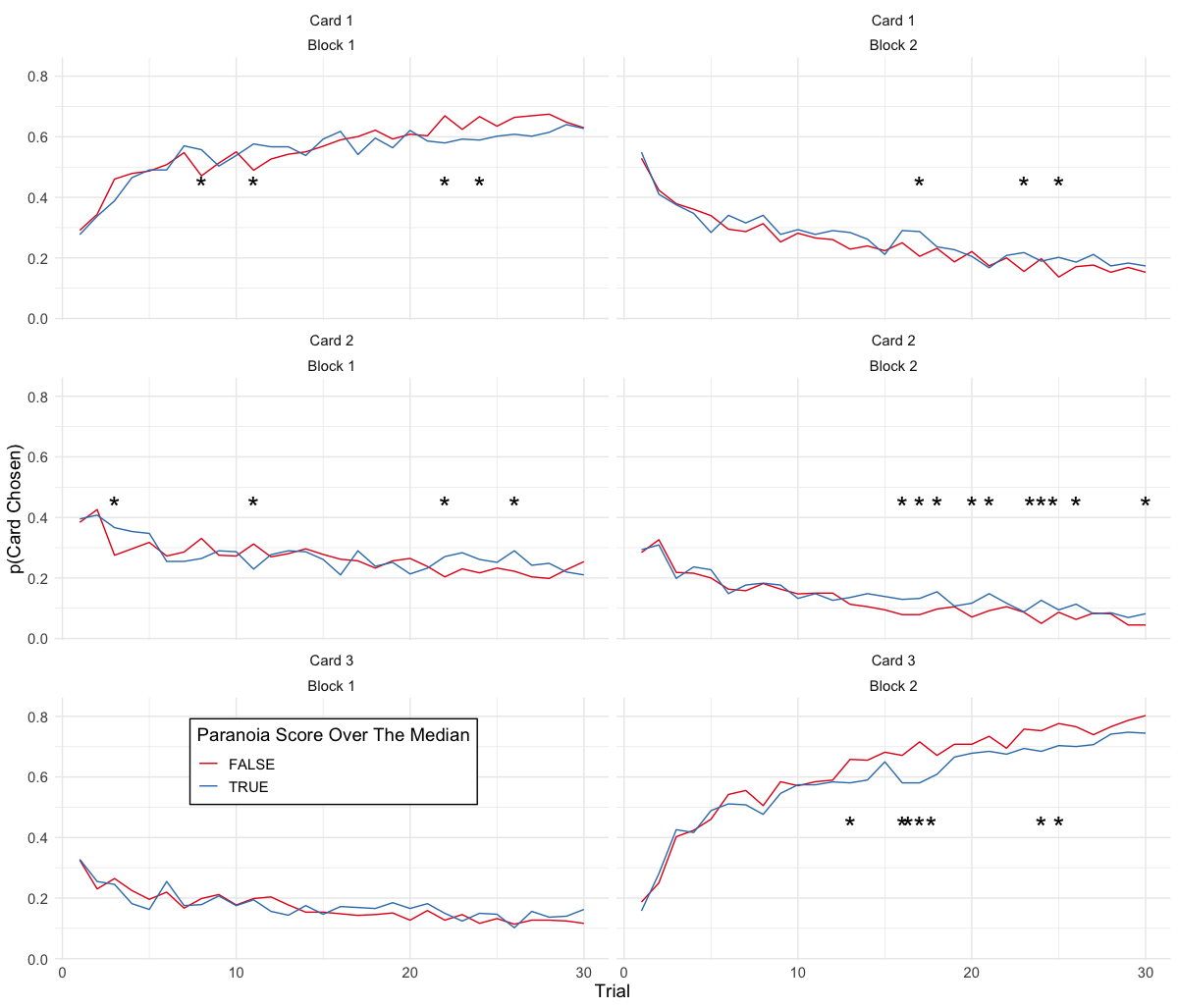
**

**Figure S2: Probability of choosing a particular card in each block for high and low paranoia.**

In Block 1, Card 1 was the optimal choice with an 80/20 probability of reward. In Block 2, Card 3 was the optimal choice, with 80/20 probability of reward. This graph demonstrates that those with higher paranoia were significantly and more consistently likely to choose the suboptimal 20/80 card (Card 2) in block two, and significantly less and more consistently likely to ignore the optimal card (Card 3) in block 2. However, those with higher paranoia were still able to learn which was the more optimal card by the end of block 2. * = p<0.05, ** = p<0.01 *** = p<0.001.
