# Supplementary material for: The computational relationship between reinforcement learning, social inference, and paranoia
Source: PLoS Comput Biol. 2022 Jul 25;18(7):e1010326. doi: 10.1371/journal.pcbi.1010326 (PMC9352206; doi:10.1371/journal.pcbi.1010326)
Supplement: S4 Fig — X = non-significant relationship. (DOCX) [file pcbi.1010326.s004.docx]

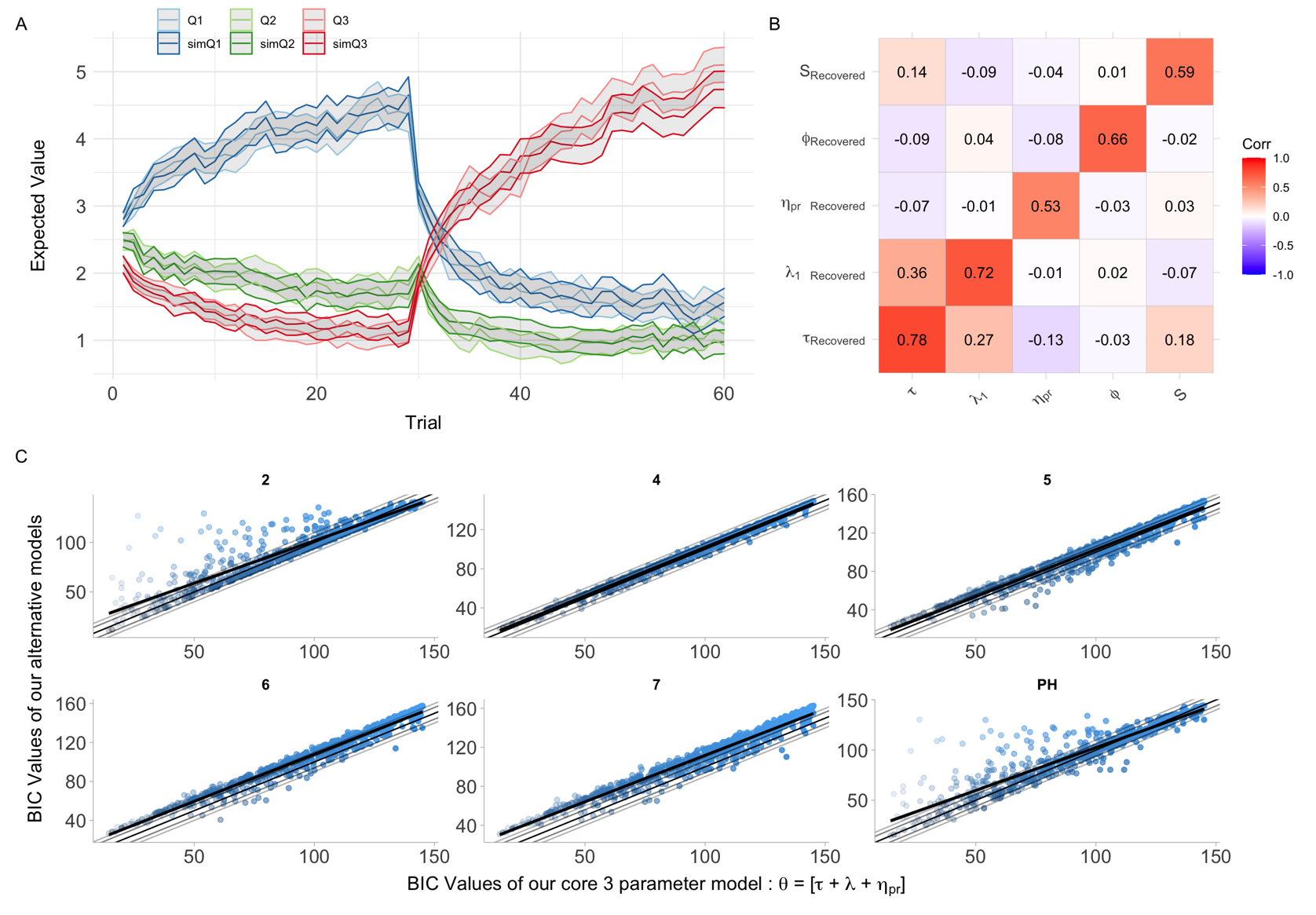
**Figure S4. Probabilistic Reversal Learning Model Fit and Recovery.**

(A) Our model was able to recapitulate the real data well. The real (Q1 – Q3) and simulated (simQ1 – simQ3) Q values generated by the model for each trial across all participants for each different symbol. (B) All parameters were recovered very well. Correlation matrix showing the Pearson correlations between the real (X axis) and recovered (Y axis) parameter. (C) The 5-parameter model produced equivalent to better BIC values compares to the 3-parameter core model. In these plots, blue dots below the line indicate better fit than the reference model (model 3) and above the line indicate the reference model fits better. Correlation comparisons between the BIC values for each alternative model (named in the facet title) and the core 3-parameter model (X axis); reference lines on each plot indicate +/- 6 and +/- 10 BIC values. Models 4 and 5 were not significantly different in individual BIC values from Model 3 (χ^2^(2) = 2.13, p = 0.345).
