# Supplementary material for: The computational relationship between reinforcement learning, social inference, and paranoia
Source: PLoS Comput Biol. 2022 Jul 25;18(7):e1010326. doi: 10.1371/journal.pcbi.1010326 (PMC9352206; doi:10.1371/journal.pcbi.1010326)
Supplement: S6 Fig — (DOCX) [file pcbi.1010326.s006.docx]

**
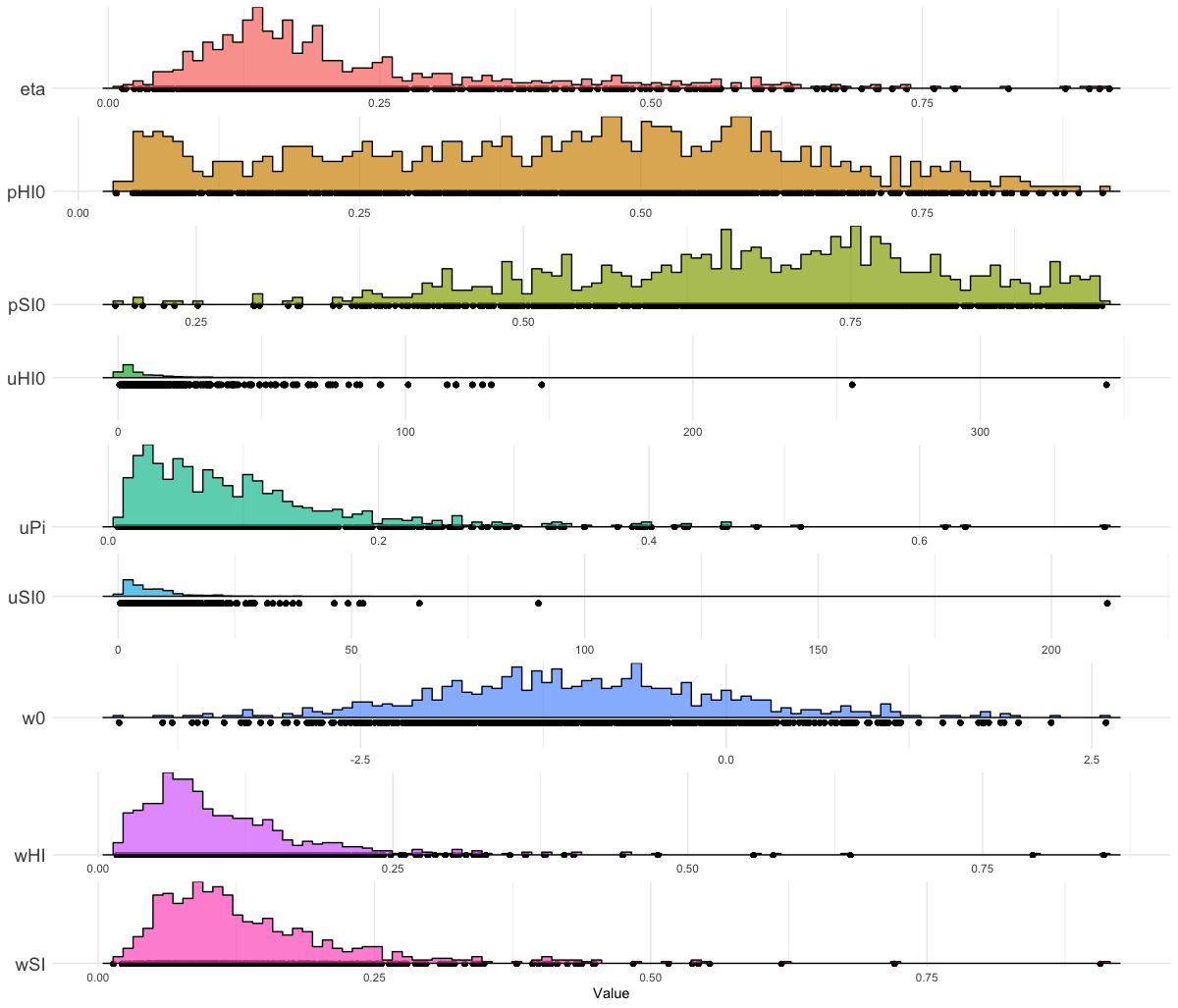
**

**Figure S6: Smoothed posterior density distributions of the individual-level fitted parameters derived from the hierarchical Bayesian fit (using CBM; modified repeated reversal Dictator Game).**
