# Supplementary material for: The computational relationship between reinforcement learning, social inference, and paranoia
Source: PLoS Comput Biol. 2022 Jul 25;18(7):e1010326. doi: 10.1371/journal.pcbi.1010326 (PMC9352206; doi:10.1371/journal.pcbi.1010326)
Supplement: S7 Fig — (A) Sum loglikelihood for each integer of pre-existing paranoia. Grey horizontal line indicates the sum loglikelihood at which the model is predicting the data by chance. (B) Sum loglikelihood for each integer of ICAR score. Grey horizontal line indicates the sum loglikelihood at which the model is predicting the data by chance. (C) Distribution of sum loglikelihood for each social condition. Grey vertical line indicates the sum loglikelihood at which the model is predicting the data by chance. (D) Correlation between real and simulated harmful intent and self-interest attributions. (E) Averaged real (grey) and simulated (coloured) harmful intent and self-interest attribution for each condition across all trials. Analysis of simulated data using a mixed effects model with ID as a random variable suggested pre-existing paranoia was positively associated with harmful intent (0.11, 95%CI: 0.05, 0.16; model S5a) but not self-interest (-0.02, 95%CI: -0.07, 0.03; model S5b), and being paired with an initially unfair Dictator did not influence harmful intent attributions, but led to larger self-interest attributions (0.27, 95%CI: 0.18, 0.37; model S4b; see S6 Fig for comparison with real data across both conditions). (DOCX) [file pcbi.1010326.s007.docx]

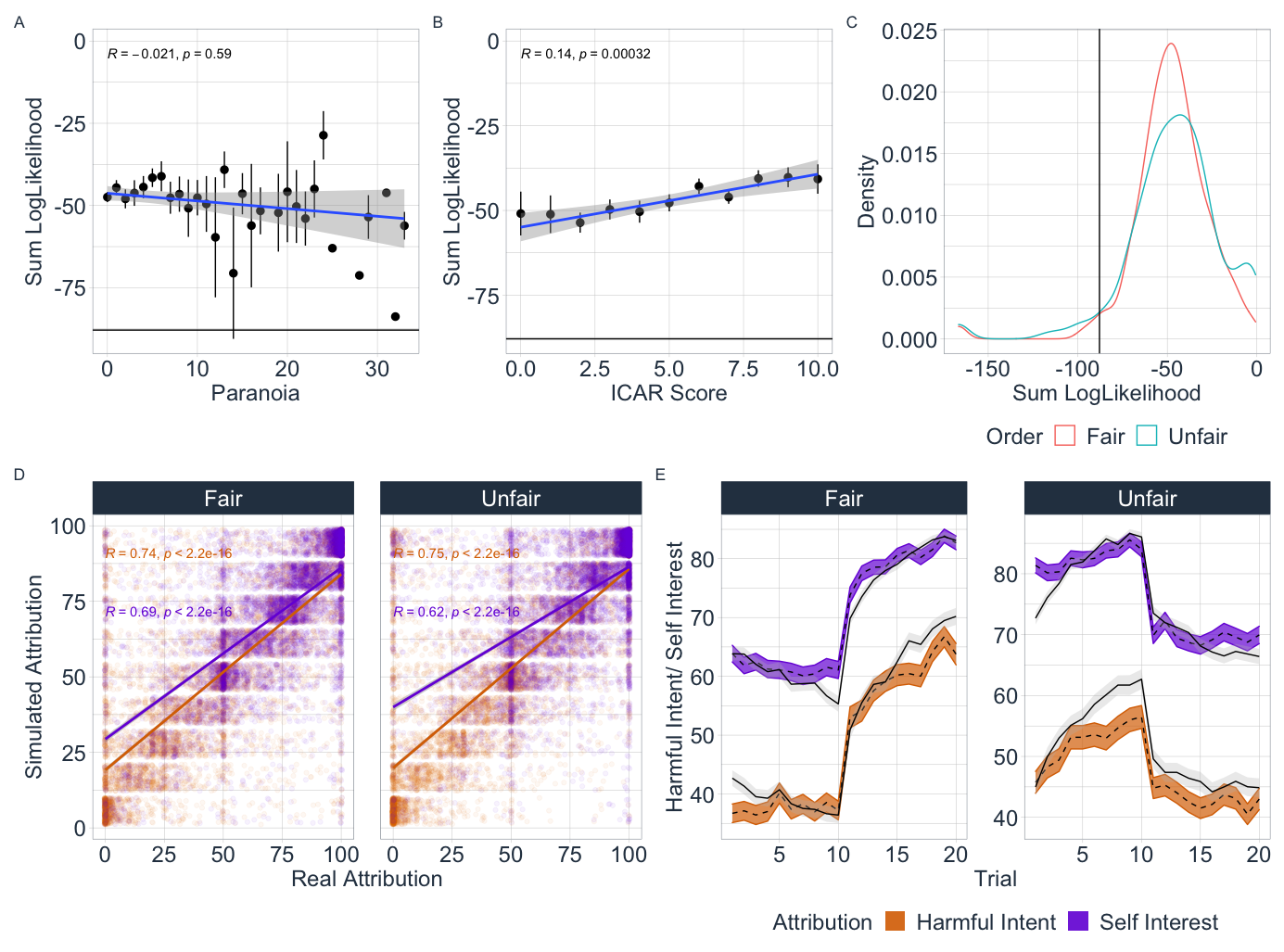
**Figure S7: Social model assessment**

(A) Sum loglikelihood for each integer of pre-existing paranoia. Grey horizontal line indicates the sum loglikelihood at which the model is predicting the data by chance. (B) Sum loglikelihood for each integer of ICAR score. Grey horizontal line indicates the sum loglikelihood at which the model is predicting the data by chance. (C) Distribution of sum loglikelihood for each social condition. Grey vertical line indicates the sum loglikelihood at which the model is predicting the data by chance. (D) Correlation between real and simulated harmful intent and self-interest attributions. (E) Averaged real (grey) and simulated (coloured) harmful intent and self-interest attribution for each condition across all trials. Analysis of simulated data using a mixed effects model with ID as a random variable suggested pre-existing paranoia was positively associated with harmful intent (0.11, 95%CI: 0.05, 0.16; model S5a) but not self-interest (-0.02, 95%CI: -0.07, 0.03; model S5b), and being paired with an initially unfair Dictator did not influence harmful intent attributions, but led to larger self-interest attributions (0.27, 95%CI: 0.18, 0.37; model S4b; see Figure S6 for comparison with real data across both conditions).
