# Supplementary material for: The computational relationship between reinforcement learning, social inference, and paranoia
Source: PLoS Comput Biol. 2022 Jul 25;18(7):e1010326. doi: 10.1371/journal.pcbi.1010326 (PMC9352206; doi:10.1371/journal.pcbi.1010326)
Supplement: S8 Fig — The 1-ηdg Bayes-Belief model (BB1eta) came first overall across the groups. Each model set was fitted using mixed-effect concurrent Bayesian modelling (Piray et al., 2018) for each group in our population. Model frequency represents the predominance of model k in the population; it is the frequency of times model k best fits all participants. Exceedance probabilities demonstrate the probability that model k is more commonly expressed than any other model in model space. Protected exceedance probabilities are more conservative as they also include the null–that no model best describes the data (Piray et al., 2018). HP = High Paranoia; HI = High ICAR score; LP = Low Paranoia; LI = Low ICAR score. (DOCX) [file pcbi.1010326.s008.docx]

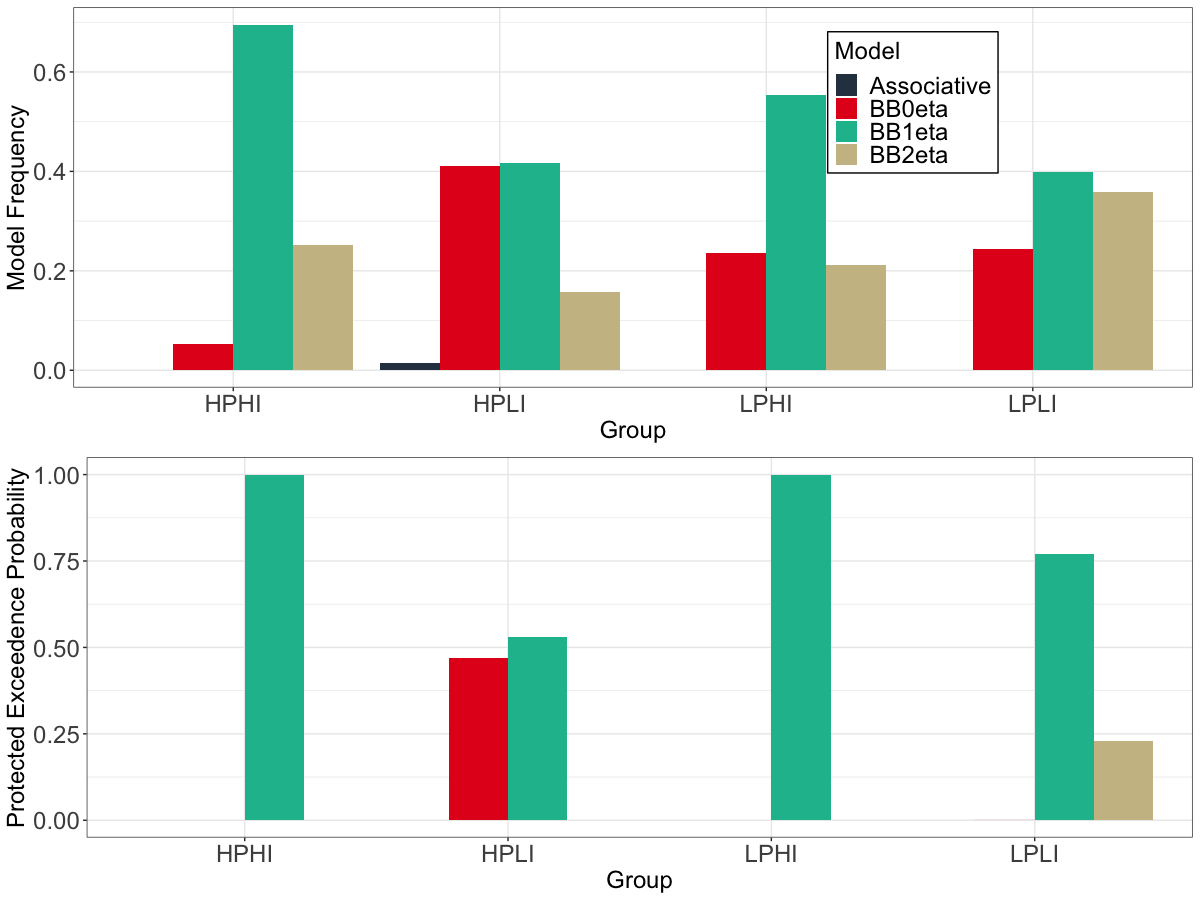


**Figure S8: Model comparison for the belief-based social model.**

The 1-η_dg_ Bayes-Belief model (BB1eta) came first overall across the groups. Each model set was fitted using mixed-effect concurrent Bayesian modelling for each group in our population. Model frequency represents the predominance of model *k* in the population; it is the frequency of times model *k* best fits all participants. Exceedance probabilities demonstrate the probability that model *k* is more commonly expressed than any other model in model space. Protected exceedance probabilities are more conservative as they also include the null – that no model best describes the data. HP = High Paranoia; HI = High ICAR score; LP = Low Paranoia; LI = Low ICAR score.
