# Supplementary material for: The computational relationship between reinforcement learning, social inference, and paranoia
Source: PLoS Comput Biol. 2022 Jul 25;18(7):e1010326. doi: 10.1371/journal.pcbi.1010326 (PMC9352206; doi:10.1371/journal.pcbi.1010326)
Supplement: S9 Fig — (A) Partial correlations between all social parameters only. (B) Partial correlations between social parameters and tau from the non-social model. (DOCX) [file pcbi.1010326.s009.docx]

**
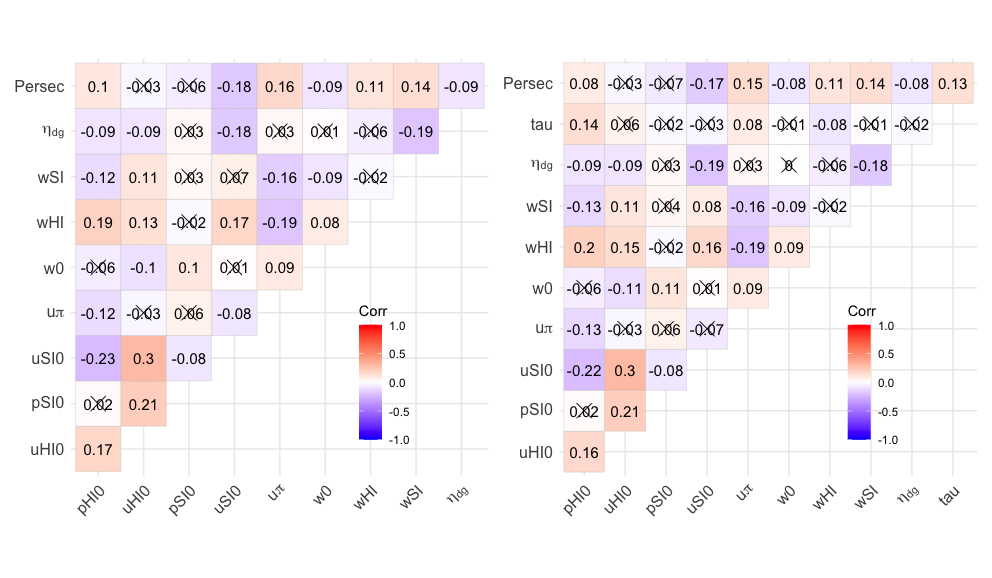
**

**Figure S9: Partial spearman correlation matrices**

(A) Partial correlations between all social parameters only. (B) Partial correlations between social parameters and tau from the non-social model.
