# Supplementary figures and images for: The computational relationship between reinforcement learning, social inference, and paranoia
Source: PLoS Comput Biol. 2022 Jul 25;18(7):e1010326. doi: 10.1371/journal.pcbi.1010326 (PMC9352206; doi:10.1371/journal.pcbi.1010326)

**
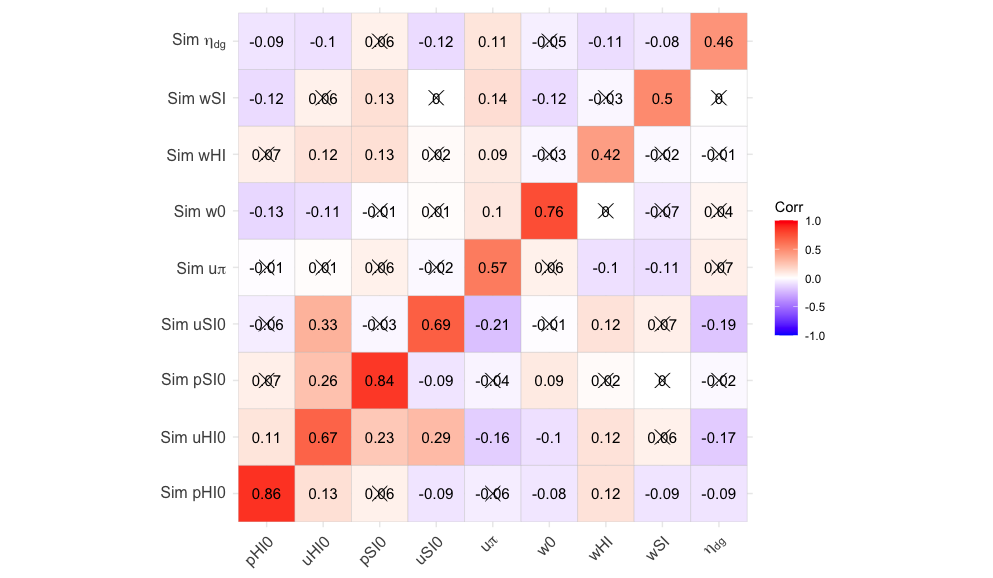
**

**Figure S10: Recovery analysis of the winning social model**

X = non-significant relationship.

Supplement: S10 Fig — X = non-significant relationship. (DOCX) [file pcbi.1010326.s010.docx]
