# Supplementary material for: The computational relationship between reinforcement learning, social inference, and paranoia
Source: PLoS Comput Biol. 2022 Jul 25;18(7):e1010326. doi: 10.1371/journal.pcbi.1010326 (PMC9352206; doi:10.1371/journal.pcbi.1010326)
Supplement: S11 Fig — (A & B) Initial policy map differences between those with high and low paranoia. Plots were constructed by using the mean w0, wSI, and wHI of those with high (persecutory ideation > 3.66) and low (persecutory ideation < 3.66) paranoid participants within our sample. Mean parameter estimates for low paranoia: w0 = -0.935, wHI = 0.102, wSI = 0.129. Mean parameter estimates for high paranoia: w0 = -1.174, wHI = 0.121, wSI = 0.158. (C) Simulated attributional changes at 10 different values (0–1) of wSI with all other parameters fixed (pHI0 = 0.5, uHI0 = 2, pSI0 = 0.5, uSI0 = 2, uPi = 2, w0 = -1, wHI = 0.1, wSI = 0.1–0.9, ηdg = 0.5). For each wSI value, 100 synthetic participants were created. (DOCX) [file pcbi.1010326.s011.docx]

**
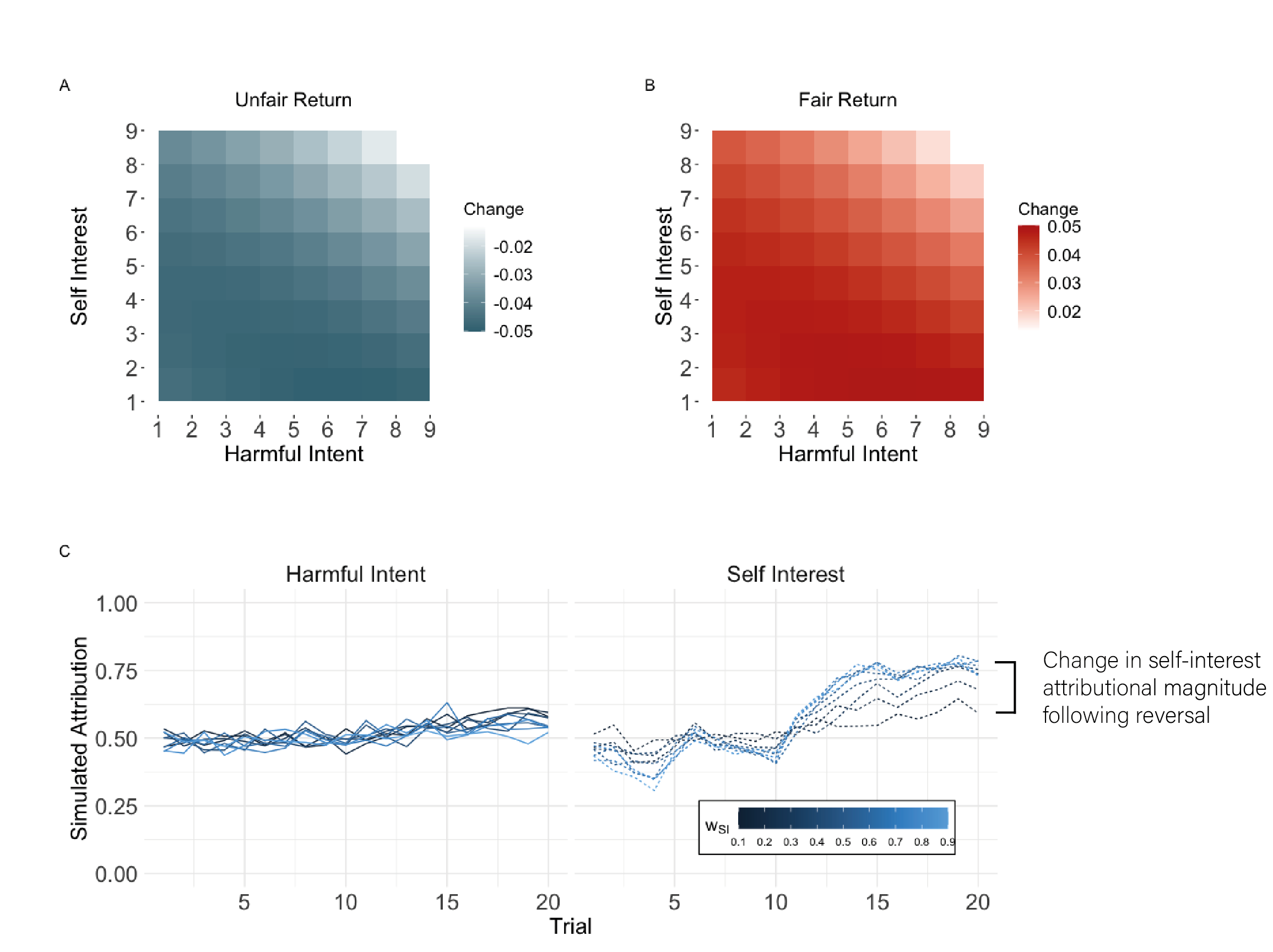
**

**Figure S11: Simulated differences of policy and attributions at several wSI values**

(A & B) Initial policy map differences between those with high and low paranoia. Plots were constructed by using the mean w0, wSI, and wHI of those with high (persecutory ideation > 3.66) and low (persecutory ideation < 3.66) paranoid participants within our sample. Mean parameter estimates for low paranoia: w0 = -0.935, wHI = 0.102, wSI = 0.129. Mean parameter estimates for high paranoia: w0 = -1.174, wHI = 0.121, wSI = 0.158. (C) Simulated attributional changes at 10 different values (0-1) of wSI with all other parameters fixed (pHI0 = 0.5, uHI0 = 2, pSI0 = 0.5, uSI0 = 2, uPi = 2, w0 = -1, wHI = 0.1, wSI = 0.1-0.9, η_dg_ = 0.5). For each wSI value, 100 synthetic participants were created.
