# Supplementary material for: The computational relationship between reinforcement learning, social inference, and paranoia
Source: PLoS Comput Biol. 2022 Jul 25;18(7):e1010326. doi: 10.1371/journal.pcbi.1010326 (PMC9352206; doi:10.1371/journal.pcbi.1010326)
Supplement: S12 Fig — (A) Our nonparanormal network replicated results from Barnby et al., (2020). (B) Stability analysis demonstrated satisfactory case-dropping estimates. (C) Bootstrapped edge weights demonstrated satisfactory estimates. See S3 Table for all edge statistics in the network. (DOCX) [file pcbi.1010326.s012.docx]

**
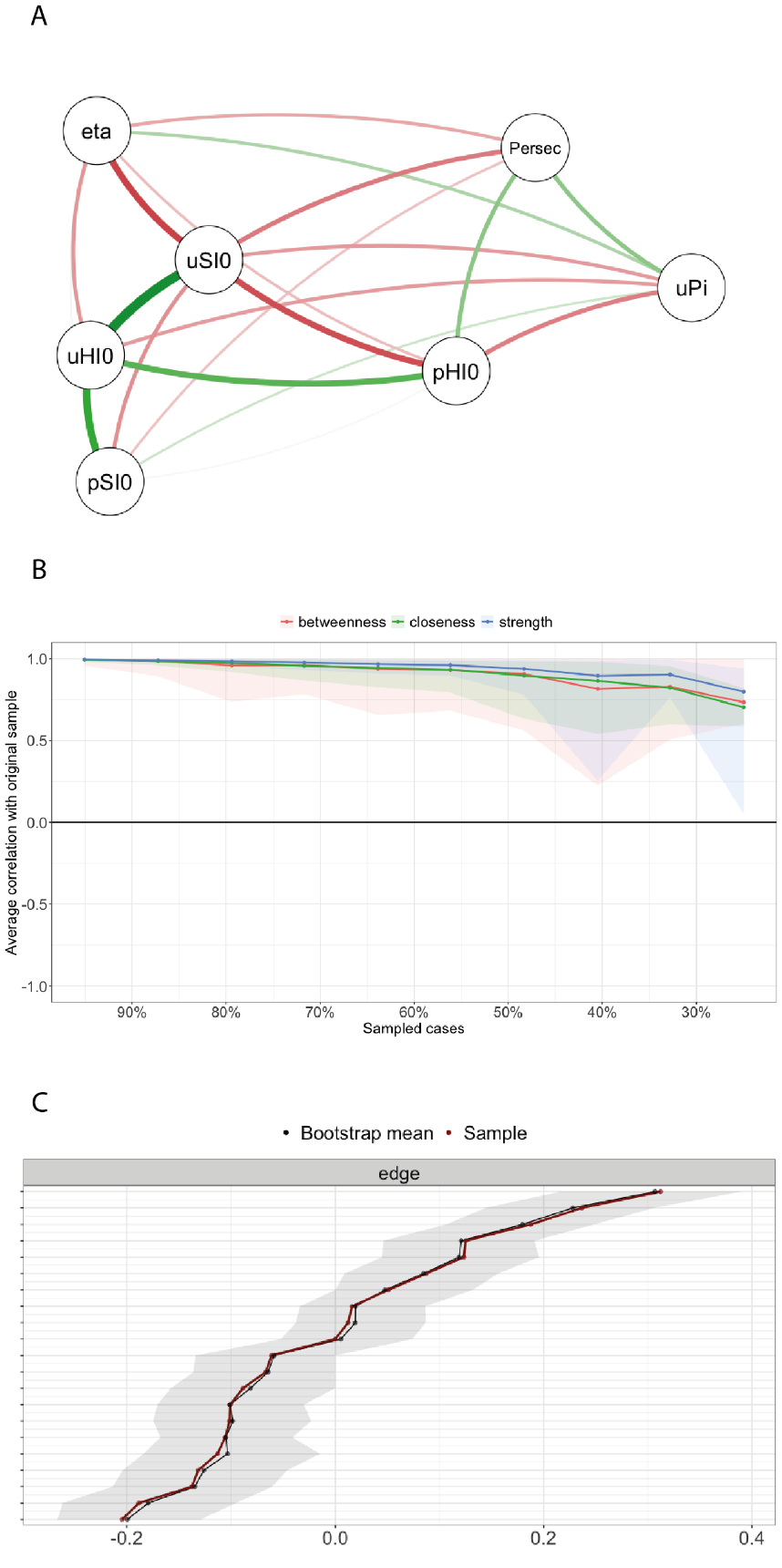
**

**S12: Network analysis between social parameters and paranoia from Barnby et al., 2020 [1]**

1. Our nonparanormal network replicated previous results. (B) Stability analysis demonstrated satisfactory case-dropping estimates. (C) Bootstrapped edge weights demonstrated satisfactory estimates. See Table S3 for all edge statistics in the network.

**Reference**

1. Barnby JM, Bell V, Mehta MA, Moutoussis M. Reduction in social learning and increased policy uncertainty about harmful intent is associated with pre-existing paranoid beliefs: Evidence from modelling a modified serial dictator game. PLoS computational biology. 2020 Oct 15;16(10):e1008372.
