# Supplementary material for: The computational relationship between reinforcement learning, social inference, and paranoia
Source: PLoS Comput Biol. 2022 Jul 25;18(7):e1010326. doi: 10.1371/journal.pcbi.1010326 (PMC9352206; doi:10.1371/journal.pcbi.1010326)
Supplement: S13 Fig — Paranoia is robustly correlated with pHI0 and uπ; the independent relationship between pHI0 and uπ may therefore be at high risk of collider bias. (DOCX) [file pcbi.1010326.s013.docx]

**
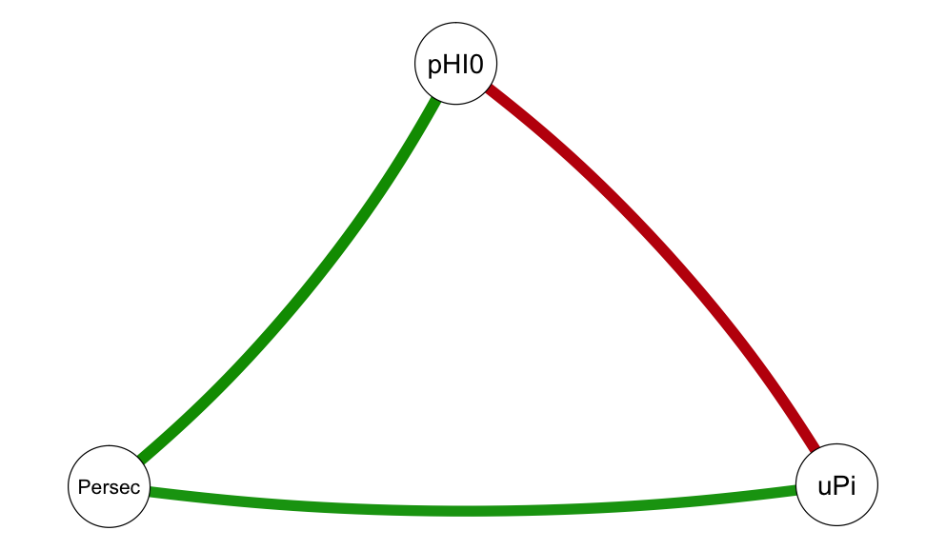
**

**Figure S13.** **Isolated network to test collider bias between nodes.**

Paranoia is robustly correlated with pHI0 and uπ; the independent relationship between pHI0 and uπ may therefore be at high risk of collider bias.
