# Supplementary material for: The computational relationship between reinforcement learning, social inference, and paranoia
Source: PLoS Comput Biol. 2022 Jul 25;18(7):e1010326. doi: 10.1371/journal.pcbi.1010326 (PMC9352206; doi:10.1371/journal.pcbi.1010326)
Supplement: S1 Table — ‘RW’ refers to the Rescorla-Wagner (RW) / Q-learning learning model. ‘PH’ refers to the Pierce-Hall salience model. ‘WS’ refers to the ‘Win-Stay; Lose-Switch’ model. (DOCX) [file pcbi.1010326.s014.docx]

**Table S1:** **Non-Social Associative Model Statistics.** ‘RW’ refers to the Rescorla-Wagner (RW) / Q-learning learning model. ‘PH’ refers to the Pierce-Hall salience model. ‘WS’ refers to the ‘Win-Stay; Lose-Switch’ model.

| **Model** | **Free Parameters** | **Log-Likelihood** | **BIC** per participant | **AIC** per participant |
| --- | --- | --- | --- | --- |
| RW | τ, λ_1_ | -43.2 | 94.6 | 90.4 |
| PH | τ, λ_1_, *S* | -41.6 | 95.4 | 89.1 |
| 3 Parameter | τ, λ_1_, η_pr_ | -39.7 | 91.7 | 85.4 |
| 4 Parameter | τ, λ_1_, η_pr,_ *S* | -38.7 | 93.7 | 85.4 |
| **5 Parameter** | **τ, λ_1_, η_pr_, *S,* ϕ** | **-36.9** | **94.6** | **84.2** |
| 6 Parameter | τ, λ_1_, η_pr_, *S,* ϕ, ζ | -36.9 | 98.4 | 85.8 |
| 7 Parameter | τ, λ_1_, η_pr_, *S,* ϕ, ζ, λ_2_ | -37.6 | 104.0 | 89.2 |
| Adapted WS | η_pr,_ *S* | -177 | 371 | 363 |
