# Supplementary material for: The computational relationship between reinforcement learning, social inference, and paranoia
Source: PLoS Comput Biol. 2022 Jul 25;18(7):e1010326. doi: 10.1371/journal.pcbi.1010326 (PMC9352206; doi:10.1371/journal.pcbi.1010326)
Supplement: S2 Table — LL, BIC, and AIC figures are indicative of the summed log probability from the combination of harmful intent and self-interest estimates for each model fitted using Maximum-A-Priori techniques. Bold highlighting represents winning models in each class. (DOCX) [file pcbi.1010326.s015.docx]

**Table S2:** **Social Model Comparison Statistics.** LL, BIC, and AIC figures are indicative of the summed log probability from the combination of harmful intent and self-interest estimates for each model fitted using Maximum-A-Priori techniques. Bold highlighting represents winning models in each class.

| **Model Type** | **Free Parameters** | **LL** | **BIC** | **AIC** |
| --- | --- | --- | --- | --- |
| Bayesian | pHI_0_, pSI_0_, uHI_0_, uSI_0_, uπ | -62.7 | 140 | 135 |
|  | pHI_0_, pSI_0_, uHI_0_, uSI_0_, uπ, η | -58.4 | 135 | 129 |
|  | pHI_0_, pSI_0_, uHI_0_, uSI_0_, uπ, η_hi_, η_si_ | -56.1 | 133 | 126 |
|  | pHI_0_, pSI_0_, uHI_0_, uSI_0_, uπ, w0, wHI, wSI | -50 | 124 | 116 |
|  | **pHI_0_, pSI_0_, uHI_0_, uSI_0_, uπ, w0, wHI, wSI, η** | **-44.2** | **115** | **106** |
|  | pHI_0_, pSI_0_, uHI_0_, uSI_0_, uπ, w0, wHI, wSI, η_hi_, η_si_ | -46.3 | 123 | 113 |
| Associative | wHI, wSI, wHI_0_, wSI_0_, λ, ESVhi_0_, ESVsi_0_, η, σ | -57.7 | 142 | 133 |
|  | **wHI, wSI, wHI_0_, wSI_0_, λ, ESV_0_, η,** σ | **-57.6** | **139** | **131** |
|  | wHI, wSI, wHI_0_, wSI_0_, λ, ESV_0_, *S*_HI_, *S*_SI_, η, σ | -65.2 | 166 | 154 |
|  | wHI, wSI, wHI_0_, wSI_0_, λ, ESV_0_, *S*_HI_, *S*_SI_, σ | -60.5 | 154 | 143 |
|  | wHI, wSI, wHI_0_, wSI_0_, λ_hi_, λ_si_, ESVhi_0_, ESVsi_0_, σ | -66 | 159 | 150 |
|  | wHI, wSI, wHI_0_, wSI_0_, λ_hi_, λ_si_, ESVhi_0_, ESVsi_0_, η, σ | -65.8 | 162 | 152 |
|  | wHI, wSI, wHI_0_, wSI_0_, λ_hi_, λ_si_, ESVhi_0_, ESVsi_0_, η_hi_, η_si_, σ | -66.4 | 166 | 155 |
