# Supplementary material for: The computational relationship between reinforcement learning, social inference, and paranoia
Source: PLoS Comput Biol. 2022 Jul 25;18(7):e1010326. doi: 10.1371/journal.pcbi.1010326 (PMC9352206; doi:10.1371/journal.pcbi.1010326)
Supplement: S3 Table — (DOCX) [file pcbi.1010326.s016.docx]

**Table S3 – Bootstrapped estimates for each edge in the replication network (Figure S12A)**

| **Edge** | **Observed mean** | **Bootstrap mean** | **sd** | **CI[lower]** | **CI[upper]** |
| --- | --- | --- | --- | --- | --- |
| **uHI_0_--uSI_0_** | **0.31** | **0.31** | **0.04** | **0.23** | **0.40** |
| **pSI_0_-- uHI_0_** | **0.24** | **0.23** | **0.04** | **0.15** | **0.32** |
| **pHI_0_-- uHI_0_** | **0.19** | **0.18** | **0.04** | **0.11** | **0.26** |
| **pHI_0_--Persec** | **0.13** | **0.12** | **0.04** | **0.05** | **0.20** |
| **uπ--Persec** | **0.12** | **0.12** | **0.04** | **0.04** | **0.20** |
| **η_dg_ -- uπ** | **0.09** | **0.08** | **0.04** | **0.01** | **0.16** |
| pSI_0_--uπ | 0.05 | 0.05 | 0.04 | -0.03 | 0.13 |
| pSI_0_--η_dg_ | 0.02 | 0.02 | 0.03 | -0.04 | 0.08 |
| pHI_0_--pSI_0_ | 0.01 | 0.02 | 0.03 | -0.05 | 0.07 |
| uHI_0_--Persec | 0.00 | 0.01 | 0.03 | -0.06 | 0.06 |
| pSI_0_--Persec | -0.06 | -0.06 | 0.04 | -0.14 | 0.02 |
| pHI_0_--η_dg_ | -0.07 | -0.06 | 0.04 | -0.14 | 0.01 |
| **η_dg_ --Persec** | **-0.09** | **-0.08** | **0.04** | **-0.17** | **-0.01** |
| **uSI_0_-- uπ** | **-0.10** | **-0.10** | **0.04** | **-0.18** | **-0.02** |
| **uHI_0_-- uπ** | **-0.10** | **-0.10** | **0.04** | **-0.17** | **-0.03** |
| **pSI_0_-- uSI_0_** | **-0.11** | **-0.10** | **0.04** | **-0.20** | **-0.03** |
| **uHI_0_--η_dg_** | **-0.11** | **-0.10** | **0.03** | **-0.17** | **-0.04** |
| **pHI_0_-- uπ** | **-0.13** | **-0.13** | **0.04** | **-0.21** | **-0.05** |
| **uSI_0_--Persec** | **-0.14** | **-0.13** | **0.04** | **-0.22** | **-0.06** |
| **pHI_0_-- uSI_0_** | **-0.19** | **-0.18** | **0.04** | **-0.27** | **-0.10** |
| **uSI_0_--η_dg_** | **-0.20** | **-0.20** | **0.03** | **-0.27** | **-0.13** |
