# Supplementary material for: The computational relationship between reinforcement learning, social inference, and paranoia
Source: PLoS Comput Biol. 2022 Jul 25;18(7):e1010326. doi: 10.1371/journal.pcbi.1010326 (PMC9352206; doi:10.1371/journal.pcbi.1010326)
Supplement: S4 Table — All regression estimates are extracted from Model J2c in the analysis code. wSI was not included in the final top model and therefore excluded from this table. (DOCX) [file pcbi.1010326.s017.docx]

**Table S4: Top Model Average of Variables Associated with decision temperature (τ)**

All regression estimates are extracted from Model J2c in the analysis code. w_SI_ was not included in the final top model and therefore excluded from this table.

| **Parameter** | **Estimate** | **Std. Error** | **95% CI** | | **Relative**  **Importance** |
| --- | --- | --- | --- | --- | --- |
|  |  |  | **lower** | **Upper** |  |
| (Intercept) | 0.07 | 0.05 | -0.02 | 0.16 |  |
| **Paranoia** | **0.11** | **0.04** | **0.03** | **0.18** | **1** |
| **Age** | **0.10** | **0.04** | **0.02** | **0.17** | **1** |
| **pHI_0_** | **0.09** | **0.04** | **0.01** | **0.16** | **1** |
| uπ | 0.00 | 0.01 | -0.06 | 0.09 | 0.09 |
| uSI_0_ | 0.00 | 0.01 | -0.09 | 0.05 | 0.10 |
| pSI_0_ | 0.00 | 0.01 | -0.10 | 0.05 | 0.10 |
| w_0_ | 0.00 | 0.01 | -0.10 | 0.05 | 0.10 |
| uHI_0_ | 0.00 | 0.01 | -0.10 | 0.05 | 0.10 |
| η_dg_ | 0.00 | 0.02 | -0.11 | 0.04 | 0.12 |
| w_HI_ | -0.05 | 0.04 | -0.14 | 0.01 | 0.85 |
| **ICAR** | **-0.10** | **0.04** | **-0.18** | **-0.02** | **1** |
| **Control** | **-0.11** | **0.04** | **-0.19** | **-0.04** | **1** |
| **Sex (Male \| Female)** | **-0.21** | **0.08** | **-0.36** | **-0.06** | **1** |
