# Supplementary material for: The computational relationship between reinforcement learning, social inference, and paranoia
Source: PLoS Comput Biol. 2022 Jul 25;18(7):e1010326. doi: 10.1371/journal.pcbi.1010326 (PMC9352206; doi:10.1371/journal.pcbi.1010326)
Supplement: S1 Text — (DOCX) [file pcbi.1010326.s018.docx]

**Text S1 Associative Social Model Formalism**

We constructed a variation on associative models previously employed to capture changes in self-esteem that, in this case, computes the subjective internal value of harmful intent and self-interest attributed to a social partner. The model at its core computes a cached Markovian value function for expected social value of a monetary split decided upon by a partner.

Much like the non-social associative model, all types of social associative models use the difference between the expected social value at the previous trial, and the feedback from the current trial to generate a social prediction error:

$${SPE}^{t}= {Social feedback}^{t}-{ESV}^{t-1}$$

Eq. 1

Where social feedback was either 0 (no money given from the partner) or 0.5 (a fair split given by the partner).

ESV was initialised using the free parameter ESV_0_ and updated according to a Rescorla-Wagner learning rule:

$${ESV}^{t}={ESV}^{t-1}+ \lambda{SPE}^{t}$$

Eq. 2

Where λ is the learning rate that captures the weight participants place upon each SPE in updating expectations for the next trial.

A simple linear map was then created between the expected social value and expected harmful intent ($\hat{HI}$)and self-interest ($\hat{SI}$) attributions at each trial, using an intercept (${wHI}_{0};{wSI}_{0}$) and weighted individually ($w_{HI}; w_{SI}$):

$$\hat{HI}^{t}={wHI}_{0}-\left( w_{HI}* {ESV}^{t} \right)$$

$$\hat{SI}^{t}={wSI}_{0}-(w_{SI}* {ESV}^{t})$$

Eq.3

To retain consistency between likelihood functions between the Bayesian Belief model and the social associative model, a binned cumulative distribution was used to assess the likelihood of each expectation against the observed data, which was itself gaussian, with the reference probability distribution summing to 1:

$${reference}_{HI}^{t}= \Phi\left( [NB, 1], {HI}^{t},\sigma\right)- \Phi\left( [0,NB], {HI}^{t},\sigma\right)$$

$${reference}_{SI}^{t}= \Phi\left( [NB, 1], {SI}^{t},\sigma\right)-\Phi\left( [0,NB], {SI}^{t},\sigma\right)$$

$${index}_{HI}^{t}=(Nb-1)* \hat{HI}^{t}+1$$

$${index}_{SI}^{t}=(Nb-1)* \hat{SI}^{t}+1$$

$$P_{HI}^{t}={reference}_{HI}^{t}({index}_{HI}^{t})$$

$$P_{SI}^{t}={reference}_{SI}^{t}({index}_{SI}^{t})$$

Eq. 4

Where *NB* is the discretised scale of harmful intent and self-interest attributions (9 bins) from 0.1 to 0.9, and HI and SI are the true harmful intent and self-interest attributions at each trial, and $\sigma$ is a free parameter that provides gaussian noise and completes the generative model.

As in the Bayesian model we also include a resetting parameter, η, that allowed flexibility to change expectations between blocks using the following convection:

$$\bar{ESV}^{t=10}={(ESV}_{0}*[1-\eta])+({ESV}^{t=10}* \eta)$$

Eq. 5

*Additional ESV parameters*

In some models, we allowed harmful intent and self-interest attributions to have their own ESV attached to them. In these cases, the followed equation would replace equation 1 and 2, respectively, and each ESV parameter would be initialised with the free parameters${ESV}_{HI}^{t=0}; {ESV}_{SI}^{t=0}$ :

$${SPE}_{HI}^{t}= Social feedback-{ESV}_{HI}^{t-1}$$

$${SPE}_{SI}^{t}= Social feedback-{ESV}_{SI}^{t-1}$$

Eq. 6

$${ESV}_{HI}^{t}= {ESV}_{HI}^{t-1}+ \lambda{SPE}_{HI}^{t}$$

$${ESV}_{SI}^{t}= {ESV}_{SI}^{t-1}+ \lambda{SPE}_{SI}^{t}$$

Eq. 7

*Additional learning rates*

In some models, we also allowed separate learning rates for each expected social value${[ESV}_{HI}^{t}; {ESV}_{SI}^{t}].$ In these cases, the following equation replaces equation 2:

$${ESV}_{HI}^{t}= {ESV}_{HI}^{t-1}+ \lambda_{HI} {SPE}_{HI}^{t}$$

$${ESV}_{SI}^{t}= {ESV}_{SI}^{t-1}+ \lambda_{SI} {SPE}_{SI}^{t}$$

Eq. 8

*Salience modifier*

To allow for Pearce-Hall like modifications within the social associative model, we also included salience parameters [$S_{HI}; S_{SI}]$ in two models which adjusted the learning rate. In these instances, the following equations replaces equation two and equation eight:

$${Sal}_{HI}^{t}=\left[ S_{HI}*\left| {SPE}_{HI} \right| \right]+\left[ \left( 1-S_{HI} \right)*{Sal}_{HI}^{t-1} \right]$$

$${Sal}_{SI}^{t}=[S_{SI}*\left| {SPE}_{SI} \right|]+[\left( 1-S_{SI} \right)*{Sal}_{SI}^{t-1}]$$

Eq. 9

$${Adjusted learning rate}_{HI}^{t}= \lambda_{HI}* {Sal}_{HI}^{t}$$

$${Adjusted learning rate}_{SI}^{t}= \lambda_{SI}* {Sal}_{SI}^{t}$$

Eq. 10

$${ESV}_{HI}^{t}= {ESV}_{HI}^{t-1}+[{Adjusted learning rate}_{HI}^{t}* {SPE}_{HI}^{t}]$$

$${ESV}_{SI}^{t}= {ESV}_{SI}^{t-1}+[{Adjusted learning rate}_{SI}^{t}* {SPE}_{SI}^{t}]$$

Eq. 11

*Dual-*η_dg_ *model*

Finally in one model we allowed two η_dg_ parameters [$\eta_{HI}; \eta_{SI}$] to reset expectations over each expectation over harmful intent and self-interest attributions. In this case, the following equation will replace equation 5:

$$\bar{ESV}_{HI}^{t=10}=\left( {[ESV}_{HI}^{t=0}*\left[ 1-\eta_{HI} \right] \right)+\left( {ESV}_{HI}^{t=10}* \eta_{HI} \right)$$

$$\bar{ESV}_{SI}^{t=10}=({[ESV}_{SI}^{t=0}*[1-\eta_{SI}])+({ESV}_{SI}^{t=10}* \eta_{SI})$$

Eq. 12
