# Supplementary material for: The computational relationship between reinforcement learning, social inference, and paranoia
Source: PLoS Comput Biol. 2022 Jul 25;18(7):e1010326. doi: 10.1371/journal.pcbi.1010326 (PMC9352206; doi:10.1371/journal.pcbi.1010326)
Supplement: Attachment — Submitted filename: R_to_R.docx [file pcbi.1010326.s019.docx]

**Dear Prof Gershman,**

**Thank you very much for your response. We were very pleased to hear that the paper was invited for resubmission following the integration of both sets of reviewer comments. In responding to the reviewers’ comments, we feel the manuscript is substantially strengthened, and for this, we are grateful.**

**We now turn to the specific reviewer comments.**

**Reviewer 1:**

**We greatly thank the reviewer for their attention to detail in the review of our manuscript, and the emphasis on the communication of our computational model-based analysis. The reviewer’s comments have led to a substantially improved manuscript. Please find our responses to each of the reviewer’s comments below in bold, and where appropriate, excerpts from the manuscript where we have applied changes.**

Major concerns

1. The current organization of the paper makes it difficult to track what is important. An additional paragraph at the beginning of the results section that summarizes the key methodology would greatly help with understanding the results without jumping around the paper to the methods.

**Thank you to the reviewer for this observation. We have now included a paragraph at the start of the results that briefly covers the core methodology of the study for clarity.**

**‘We administered a non-social probabilistic reversal learning paradigm and a modified repeated Dictator Game to 693 participants, in addition to collecting data on participants persecutory ideation (hereafter termed ‘paranoia’; measured via subscale B of the Revised Green Paranoid Thoughts Scale; R-GPTS, Freeman et al., 2021), general cognitive ability (using the International Cognitive Ability Resource – Progressive Matrices [ICAR]; Condon & Revelle, 2014), age, sex, and task comprehension. We conducted computational model-agnostic and model-based analyses; in model-based analyses, we tested a range of associative models for the non-social task (k=8), and a range of associative (k=7) and Bayesian-belief (k=6) models in the social task to account for participant choice and attributional behaviour, respectively. In addition to reporting model-based and model-agnostic outcomes for each paradigm, we report the relationship between key parameters across winning non-social and social computational models (see Figure 1 and Methods for more details).’**

2. I am having trouble following the models as the document is currently written. Are there terms that can be applied to the symbols described to increase clarity instead of just symbols? Furthermore, in the discussion, you don’t cite the symbols, which makes it even more confusing to switch from only symbols to only text. Please review the results and discussion section for consistency in language.

**We apologise that the model nomenclature was confusing and welcome the opportunity to clarify. We have now ensured all parameter symbols are appropriately described when introduced in the text and figure legends, and that the description of parameters is kept uniform across the entire manuscript. We have also adjusted the wording in Tables 1 and 2 to include the description of each parameter next to the appropriate symbol as a form of reference for the entire manuscript.**

3. The use of symbols without defining the meaning is very confusing; even more confusing is that some symbols for parameters never get defined (for example, pi). Figure 2 partially explains the parameters as they fit in the model but would benefit from clearer labels or text in the figure description.

**Consistent with our response above we have used the results tables to outline the parameter labels and have changed all references to parameters in the text to their descriptive names, e.g., ‘uncertainty over partner policies’, rather than just ‘up’. We have also adjusted the legend of Figure 2 to fully describe the parameters. We hope this makes the text much clearer.**

**“White nodes represent free parameters of the model. Grey shaded nodes represent numerical probability matrices built from free parameters. Thick solid and thick dotted lines represent transitions between trials. Thin solid lines represent the causal influence of a node on another node or variable. The agent or participant updates their initial beliefs (starting prior) about the partner’s intentions [p(HI, SI)^t=0^] each trial using their policy matrix of the partner [π_gen_] which maps the likelihood between a partner’s return to the participant and the partner’s true intentions weighted by three free parameters: a policy-map intercept (w_0_), sensitivity to update self-interest attributions (w_SI_), and sensitivity to update harmful intent attributions (w_HI_). The integration between the likelihood and prior belief from the previous trial is also subject to another free parameter, uncertainty over partner policies (uπ). We assume that upon detecting a change (in this task, a reversal), participants re-set their beliefs, using their priors about people in general (thin dotted line), biased by what they have learnt already about their present partner (reset-at-reversal - η_dg_). Both the policy matrix and initial beliefs about the partner are numerical matrices that assigned probabilities to each grid point of values of harmful intent (0-1) and self-interest (0-1). The model can be used to simulate observed attributions of intent given a series of returns, or inverted to infer the parameter values for participants, using experimentally observed attributions.”**

4. In S4, it seems like the blue and green lines do not map well to the same cards in the simulation in phase 2, suggesting it is not a great fit for the data. Could you please clarify?

**We thank the reviewer for pointing out this discrepancy and welcome the chance to revisit the analysis.**

**Upon review of the code, we noticed that the model pipeline was mixing simulations of Card 1 with Card 2 due to a small bug in the reward matrix. Therefore, in the original graph, simulations were confusing Card 1 with Card 2 after reversals and therefore looked to be tracking different Q values to the true data fitted by model inversion.**

**Nevertheless, our simulations would not have affected the fit of models to the data during model comparison and selection; the inverted models were agnostic to the simulated data produced from the models and did not rely on a generative reward matrix (rewards were drawn from true rewards obtained by participants). Likewise, this discrepancy would not have influenced the regression model metrics which were run on fitted (not simulated) data. Rather, this confusion in the simulated data would have only affected the model recovery of participant parameters and Figure S4A.**

**We have now corrected the reward matrix in the simulated, generative portion of the model and recreated Figure S4A. In addition, we have rerun the model recovery of our winning model and found that recovery correlations are superior to our previous iteration (Figure S4B). Finally, we have updated our codebase on GitHub to register this correction.**

5. From the introduction, abstract, and title, I assumed that the comparison of reinforcement modelling in the non-social task with the social intent parameters was a major result of the paper, but the actual result comparing the two models is brief. Would it be possible to include Figure S9 in the main text to visualize this finding more clearly?

**Thank you for this suggestion. We have now moved Figure S9 to the main text to centralise this finding. We have also combined it with the permutation analysis in Figure S10 to demonstrate the significant correlations of all social parameters against tau for completeness.**

**“Figure 3. The relationship between decision temperature, attributions, and social task parameters. (A) Spearman correlations between decision temperature and mean attributions observed summed across 20 trials for each participant. (B) Permutation analysis of the relationship between decision temperature, and computational model-based parameters from the winning model and pre-existing paranoia. The grey distribution represents the null distribution following random sampling of the population for each Spearman pairwise correlation. The true Spearman correlations of each social parameters against tau are depicted for each parameter. Only the strength of prior beliefs over harmful intent (pHI_0_**$\boldsymbol{;}\boldsymbol{\rho}$ **= 0.16,** $\boldsymbol{p}_{\boldsymbol{permuted}}$ **~ 0), uncertainty over partner policies (uπ;** $\boldsymbol{\rho}$ **= 0.09,** $\boldsymbol{p}_{\boldsymbol{permuted}}$ **= 0.015), and paranoia (**$\boldsymbol{\rho}$ **= 0.16,** $\boldsymbol{p}_{\boldsymbol{permuted}}$ **~ 0) were associated with decision temperature. Red lines denote that the observed correlation with tau is very unlikely due to chance (p < 0.05). Black lines denote the observed correlation is more likely due to chance (p > 0.05).”**

Smaller comments

1. Not all acronyms are defined, making it difficult to keep track of what is important. ICAR is one example. Other examples are HI and SI. I figured out what they mean by context, but these acronyms mean something different to me (homicidal and suicidal ideation), which led to additional confusion. Please define all acronyms.

**Thank you for this comment and for pointing out areas where greater clarity is needed. We have now ensured all acronyms are defined where they first appear and are consistent throughout the manuscript. To speak to your specific examples:**

**“Persecutory ideation (hereafter termed ‘paranoia’; measured via subscale B of the Revised Green Paranoid Thoughts Scale; R-GPTS, Freeman et al., 2021), general cognitive ability (using the International Cognitive Ability Resource – Progressive Matrices [ICAR]; Condon & Revelle, 2014), age, sex, and task comprehension.”**

**“In brief, after controlling for confounders, paranoia was associated with larger and less flexible harmful intent attributions (HI). Paranoia did not influence self-interest attributions (SI).”**

2. Why is eta sometimes written out and sometimes a symbol? Are they different?

**We thank the reviewer for this comment and apologise for the confusion. There are two ‘eta’ parameters in this paper, one to allow for a change-point in the non-social task and another to allow for a change-point in the social task. We have removed all instances of ‘eta’ and replaced it with the specific appropriate parameter symbol (with description) depending on the model:**

**“We found that a modified Pearce-Hall model including a ‘reset-at-reversal’ parameter (η_pr_) best accounted for the data while retaining rich enough a parametrization to allow straightforward comparisons across individuals (see methods for full model comparison statistics, equations, and model fitting procedure; Table S1).”**

**“We also found a positive relationship between uncertainty over a partner’s policy and how much participant’s reset their beliefs following a reversal (η_dg_; 0.09, 95%CI: 0.01, 0.16; See Figure S12a; Table S3).”**

3. I found the abstract was misleading about the sample size and recommend changing it to the size of the final dataset used.

**We thank the reviewer for the comment and apologise that we appear misleading in the abstract. We have now adjusted the *n* in line with our final sample size.**

**“We tested whether the same population (n=693) responded similarly to non-social and social contingency changes in a probabilistic reversal learning task and a modified repeated reversal Dictator game, and the influence of paranoia on both.”**

4. Typo pg 27 - “A simulated annealing approach on parameter values was combined was followed by gradient-ascent on MAP to minimize the chance of missing important MAP maxima.”

**We have now corrected this error.**

5. Typo pg 15 - “Larger priors beliefs”

**We have now corrected this error.**

6. Typo pg 14 - “optimal choices are not ignored in those who are more paranoid, optimal choices may instead take longer on average to become exploited, leaving more room for ambiguity.”

**We have now corrected this error.**

7. I found this sentence in the abstract confusing. Consider rewriting: "Consistent with prior work we show that, after reversals, pre-existing paranoia was independently associated with uncertainty around a partner’s behavioural policy and rigidity in harmful intent attributions in the social task, and in the probabilistic task, larger decision temperatures and commitment to suboptimal cards."

**Thank you for this comment and the opportunity to be clearer - we have now reworded this sentence:**

**“Consistent with prior work we show that pre-existing paranoia was associated with uncertainty around a partner’s behavioural policy and rigidity in harmful intent attributions in the social task. In the non-social task we found that pre-existing paranoia was associated with larger decision temperatures and commitment to suboptimal cards.”**

8. I appreciated the summary of the findings at the end of sections, for example on the middle of page 8. It may be helpful to implement this elsewhere, as there is a lot of repetition when looking at results with and without confounders that makes it harder to track the key findings.

**We appreciate the reviewer’s comment and welcome a chance to be clearer in the reporting of our results. We have now included summarising statements at the beginning of each subsection for model-based and model-agnostic analyses:**

**‘In sum, after controlling for confounders, paranoia was positively associated with choosing the worst card following a reversal. Paranoia was only associated with earning fewer rewards and win-switch biases following reversals. Paranoia was not associated with less accurate forced-choice self-reports asking which was the best card.”**

**‘In brief, after controlling for confounders, paranoia was associated with larger and less flexible harmful intent attributions (HI). Paranoia did not influence self-interest attributions (SI).’**

**“As an overview, we found that paranoia was only associated with decision temperature (τ) and absolute trial-wise prediction errors after adjusting for confounders.”**

**“To outline, data was best explained by a Bayesian-Belief model that hypothesised that participants’ separately weight changes to harmful intent and self-interest attributions following changes to a partner’s behaviour. After adjusting for confounders, paranoia was associated with greater uncertainties over a partner’s policy (uπ), stronger priors over harmful intent (pHI_0_; but not self-interest, pSI_0_), and a higher sensitivity to explain changes in behaviour by adjusting attributions about a partner’s self-interest (w_SI_), but not adjustments to harmful intent (w_HI_).”**

**“Finally, we examined the relationship between derived parameters that shared independent relationships with paranoia across both tasks. In brief, we found that decision temperature (τ) was positively associated with HI (but not SI), and positively associated with the strength of priors over harmful intent of the partner (pHI_0_).”**

9. I appreciate the use of preregistration and making your code available.

**Thank you very much.**

**Reviewer 2:**

**We thank reviewer 2 for their comments regarding the interpretation of our article, as well as the past evidence we include. We have adjusted the manuscript in several ways following their points and suggestions.**

The authors prefer to imply that everything is social, rather than appealing to the perhaps more parsimonious suggestion that their results reflect a simpler non-social learning mechanism in play in both their social and non-social tasks.

I am not sure that this approach could have given them the dissociation that they claim. Perhaps if they had shown no relationship between paranoia and reversal learning they would be correct to center the social. As it stands, the result is ambiguous and open to interpretation.

Their all-social interpretation seems to stem from the assymmetry towards the social in their social task. Could it be though that the social task is simply more complex and recursive and more demanding of a general learning mechanism than their non-social reversal task?

**We thank the reviewer for their observation, although having carefully looked at our paper we cannot see where we claim that everything is social. Instead, we aim to bridge non-social and social mechanisms. We are not trying to claim one is less important than the other. If anything, we argue for a general experience of uncertainty that relates to some social outcomes. Following the comments from this reviewer and congenial with suggestions from reviewer 1, we have now made the relationship between the social and non-social more prominent by the inclusion of Figure 3.**

**We would also like to point the reviewer to the following examples where we actively avoid making clear social/non-social distinctions.**

**From the abstract, we suggest a common mechanism which is expressed asymmetrically in different contexts:**

**“Our results converge across both classes of model, suggesting paranoia is associated with a general uncertainty over the state of the world (and agents within it) that takes longer to resolve, although we demonstrate that this uncertainty is expressed asymmetrically in social contexts.”**

**In the introduction, we put forward rigid updating of harmful intent irrespective of the partner’s style of behavioural change:**

**‘In line with prior evidence, we predicted that during the probabilistic reversal learning task paranoia would be associated with lower decision consistency, greater win-switch rates, and greater perseveration errors following the reversal. In the modified repeated reversal Dictator game, we hypothesised that higher paranoia would lead to rigidity in harmful intent attributions formed about a partner when a partner’s behaviour changes, regardless of whether they were fair or unfair pre-reversal.”**

**In the discussion, we suggest that our results bridge between general learning mechanisms and specific social contexts:**

**‘Our model and data raise hypotheses that may bridge general reinforcement learning and specific phenomenological explanations of the paranoia and allow experimental testing of predictions with formalised computational targets.’**

**‘‘First, there may be a common biological mechanism responsible for the expression of uncertainty in both non-social and social contexts.’**

I would suggest that they temper the "all social" interpretation, and perhaps look to future studies to disambiguate more decisively. For example, Lockwood et al suggest that a social brain explanation of some phenomenon might be more warranted if the social computations are realized neurally in different structures or circuits: <https://www.cell.com/trends/cognitive-sciences/fulltext/S1364-6613(20)30168-6>

Recent meta-analysis of social and non-social prediction error studies did identify a set of common and relatively unique regions (including the DMPFC) where social computations might be implemented (<https://pubmed.ncbi.nlm.nih.gov/35017672/>). It strikes me that these predictions about implementation might be less ambiguous than comparisons of task behaviours.

**We thank the reviewer for this suggestion and agree with the Lockwood et al., paper (2020) that neural localisation may offer an additional way to characterise neurocognitive mechanisms preferentially used for social inference.**

**We have added this statement in the discussion to account for the reviewer’s suggestion:**

**“On a neural there is evidence that social context may be biologically realised through the engagement of different structures (Lockwood et al., 2020), including the dorsomedial prefrontal cortex where social computations may be implemented (Diaconescu et al., 2020). We predict that dopaminergic changes that underlying learning in multiple contexts may lead to context specific effects (e.g., social vs non-social learning) such as a participant’s uncertainty over their partner (uπ).”**

There are also some minor issues with scholarship and citation. They suggest that Reed et al included a social task, they did not. They fail to acknowledge that Suthaharan et al compared social and non-social Probabilistic reversal learning and failed to find a differential relationship with paranoia.

**We thank the reviewer for this observation. We indeed did mistakenly attribute the social task to Reed and have now corrected this to the Suthaharan, 2021 paper. We have also noted the previous finding from the Suthaharan paper:**

**“An alternative would be to create a ‘social’ version of a non-social task (e.g., Suthaharan et al., 2021). Suthaharan and colleagues (2021) aimed to assess whether probabilistic reversal learning in those with higher paranoia differed between card decks that were and were not putatively controlled by a social agent, finding no difference in parameter estimates in those more paranoid across both tasks. However, tasks such as that used by Suthaharan and colleagues may be measuring social observation more than they are measuring social interaction; the latter requires an interaction partner’s behaviour to be ‘online’ (i.e., the decisions of the partner result in outcomes for both the partner and the participant; Schilbach, 2014).”**

They could also focus more on studies that have employed tasks in which the social and non-social are pitted against each other in the same task. Diaconescu et al springs to mind. There no specific effect of schizophrenia was found on the social aspect of the task (though no paranoia association was reported, we might imagine that SZ patients would be more paranoid, since 90% of them report paranoia).

**We thank the reviewer for this suggestion. We are not sure which A. Diaconescu study they refer to, but we already cite work from this lab (Diaconescu et al., 2020; Henco et al., 2020) comparing non-social and social learning in those with schizophrenia and borderline personality disorder in our introduction and discussion:**

**‘To examine reinforcement learning observations within social contexts relevant to paranoia, experimenters have also framed probabilistic tasks in terms of interaction with social agents, demonstrating that those with higher paranoia are slower learners and more sensitive to changes in the social environment (Henco et al., 2020), more rigid in their beliefs about partners (Wellstein et al., 2020), and less likely to take advice from partners (Diaconescu et al., 2020; Hertz et al., 2021).’**

**‘Given the empirical relationship between pre-existing paranoid beliefs and psychosis on uncertainty over environments (Adams et al., 2018; Deserno et al., 2020; Henco et al., 2020; Moutoussis et al., 2011; Reed et al., 2020; Sterzer et al., 2018; Suthaharan et al., 2021) it is unsurprising that both non-social and social uncertainties are jointly related to paranoia in this present experiment, although we demonstrate this explicitly in relation to pre-existing paranoia and attributions in the moment.’**

**Nevertheless, we additionally include an extra citation of the Diaconescu study (**[**https://elifesciences.org/articles/54051**](https://elifesciences.org/articles/54051)**) in the discussion speaking to the arbitration between individual and social learning:**

**“On a neural there is evidence that social context may be biologically realised through the engagement of different structures (Lockwood et al., 2020), including the dorsomedial prefrontal cortex where social computations may be implemented (Diaconescu et al., 2020). We predict that dopaminergic changes that underlying learning in multiple contexts may lead to context specific effects (e.g., social vs non-social learning) such as a participant’s uncertainty over their partner (uπ).”**

Also conspicuous in its absence is a treatment of Rossi-Goldthorpe et al - where a manipulation of social group identity changed prior beliefs but did not interact with paranoia - instead paranoia was more strongly related to non-social aspects of the task and computational model (paranoid participants inferred that their own task performance was unreliable).

**We thank the reviewer for this suggestion. We have now included the Rossi-Goldthorpe reference in the discussion:**

**“First, uncertainty over others or over the self as a prerequisite for persecutory ideation has been theoretically (Bentall et al., 2001; Diaconescu et al., 2019; Freeman, 2016; Fletcher & Frith, 2009) and empirically (Henco et al., 2020; Rossi-Goldthorpe et al., 2021; Salvatore et al., 2012) supported.”**

I also feel its a little inaccurate and overly simplistic to appeal to glutamate mediating priors and dopamine relatng to social processing.

The authors might consider these this paper when unpacking their results in the context of neurochemistry:

<https://pubmed.ncbi.nlm.nih.gov/35289748/>

These data suggest that the form of what is being learned and the contingencies (whether social or non-social) are crucial to the impact of dopamine manipulations.

**We have removed the mention of glutamate in our discussion, and additionally included mention of the suggested study looking at primary vs secondary learning:**

**“Prior theoretical work explains the relationship between dopamine (dys)regulation, psychosis, and probabilistic reasoning (Fletcher & Frith, 2009; Howes & Kapur, 2009), and empirical evidence has supported the common role of dopamine (dys)regulation in influencing uncertainty about the world (Adams et al., 2020; Reed et al., 2020), the learning of information from primary vs secondary sources (Rybicki et al., 2022), adjusting harmful intent and externalising attributions (Barnby et al., 2020b; Newman-Taylor et al., 2020), and increasing psychotic experiences (Nour et al., 2018; Voce et al., 2019).”**
